# A biomimetic nanofluidic diode based on surface-modified polymeric carbon nitride nanotubes

**DOI:** 10.3762/bjnano.10.130

**Published:** 2019-06-27

**Authors:** Kai Xiao, Baris Kumru, Lu Chen, Lei Jiang, Bernhard V K J Schmidt, Markus Antonietti

**Affiliations:** 1 Max Planck Institute of Colloids and Interfaces, Department of Colloid Chemistry, 14476 Potsdam, Germany; 2 Key Laboratory of Bio-inspired Smart Interfacial Science and Technology of Ministry of Education, School of Chemistry, Beihang University, 100191 Beijing, P.R. China

**Keywords:** carbon nitride, ion transport, nanochannel, nanofluidic system, photofunctionalization

## Abstract

A controllable ion transport including ion selectivity and ion rectification across nanochannels or porous membranes is of great importance because of potential applications ranging from biosensing to energy conversion. Here, a nanofluidic ion diode was realized by modifying carbon nitride nanotubes with different molecules yielding an asymmetric surface charge that allows for ion rectification. With the advantages of low-cost, thermal and mechanical robustness, and simple fabrication process, carbon nitride nanotubes with ion rectification have the potential to be used in salinity-gradient energy conversion and ion sensor systems.

## Introduction

Ion transport is the basis of energy and sensory systems in living organisms [1]. All biological signal transport and transduction processes, including pain, haptics, vision, audition, olfaction, and muscular movement, as well as energy conversion and consumption are associated with ion transport [2–3]. For example, a plant injured on one leaf by a nibbling insect can alert its other leaves to begin anticipatory defense responses by Ca^2+^ ion transport [4]. A very significant ion-transport mechanism based on Na^+^ and K^+^ across cell membranes results in the generation of the action potential, which plays a crucial role in the sensory system of intelligent life [5]. In the process of photosynthesis, light-driven passive ion transport results in a proton gradient across cell membranes, which enables the production of adenosine triphosphate (ATP) via ATP synthase [6]. All these passive and active ion transport processes in vivo occur in biological protein nanopores, which are very fragile. Therefore, it is challenging to reproduce a similar ion transport in vitro [7]. In the last decades, scientists from chemical and material fields have attempted to achieve similar ion transport in solid-state nanopores or nanochannel systems, and other applied technologies [8–11].

The ion transport in solid-state materials has been studied with various nanostructures, i.e., 1D nanopores/nanochannels/nanotubes, 2D layered membranes, and 3D porous membranes, which can be fabricated from inorganic, organic, and polymer materials [12–13]. To date, three main ion-transport properties, namely, ion selectivity, ion rectification and ion pumping of biological ion channels have been successfully achieved in solid-state materials [14–16]. All these controllable active and passive ion transport mechanisms are based on the electrical double layer (EDL) effect [17–18]. In a charged nanochannel, only one ionic component can be transported across the nanochannel when the diameter of the nanochannel matches or falls below the Debye screening length because of the electrostatic interactions between the ions and the charged channel walls. As a result, positively charged nanochannels preferentially transport anions instead of cations, while the negatively charged nanochannels selectively transport cations [19–21]. This is the origin of ion selectivity. To realize ion rectification, asymmetric nanochannels and/or asymmetric surface-charge distributions are needed. In this case, the selected ions will be transported preferentially from one to the other side, which is the origin of ion rectification (or ion-diodes) [22]. Rectified ion transport is highly desired because it can suppress ion transport in unspecific directions, which plays a crucial role in accurate sensory systems and the generation of blue energy from salinity gradients [23–24].

We fabricated a carbon nitride nanotube membrane (CNNM) via an anodic aluminium oxide (AAO)-templated vapor deposition–polymerization process. Subsequently, the CNNMs were modified with 3-allyloxy-2-hydroxy-1-propanesulfonic acid or allylamine through a unilateral photo-functionalization process. The photo-functionalization allows for the spatial control over the process and, hence, the introduction of a gradient of charged grafted molecules. Thus, asymmetric membranes are formed and ion-diode properties are obtained.

## Results and Discussion

### Fabrication of carbon nitride nanotube membrane

**Figure 1:**
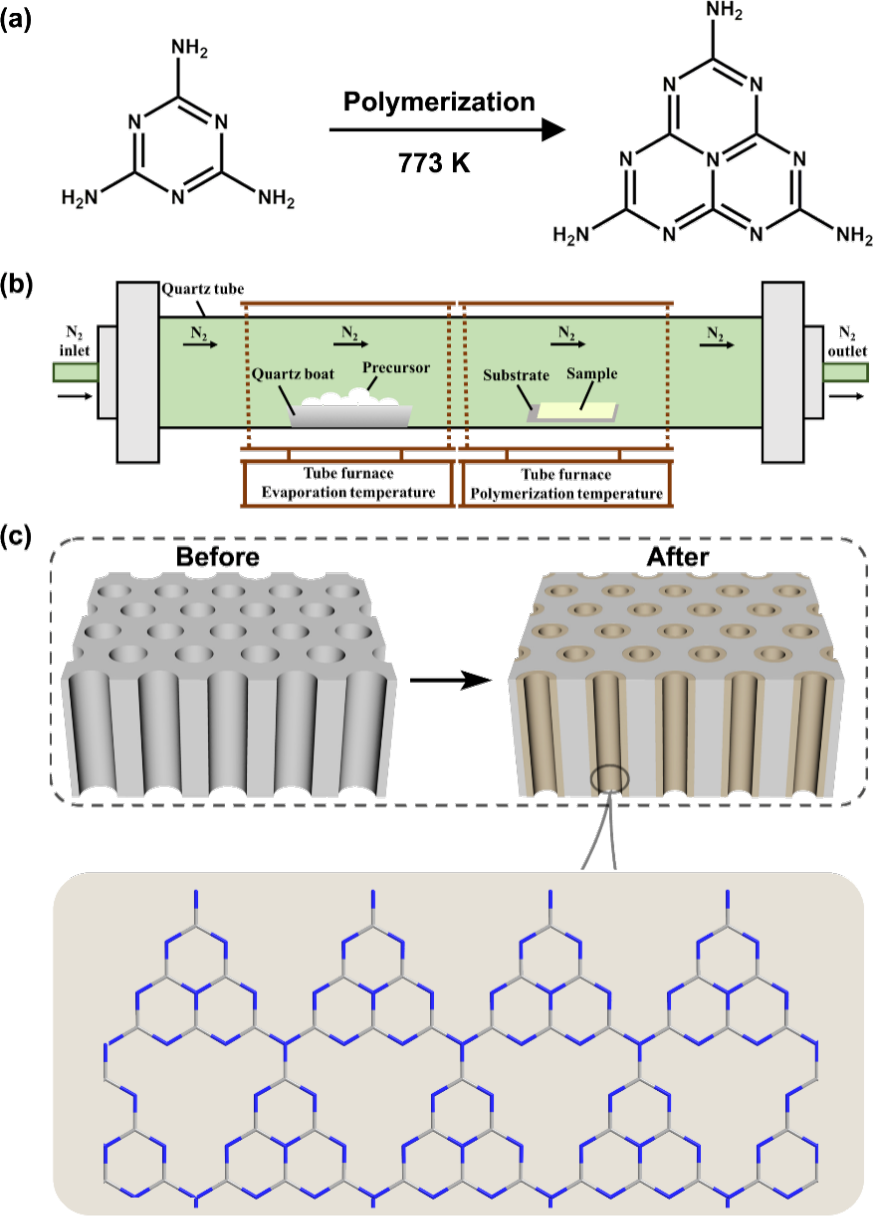
Fabrication process of carbon nitride nanotubes. (a) Synthetic route of polymeric carbon nitride nanotubes. (b) Schematic illustration of the fabrication process by vapor deposition–polymerization. (c) Schematic diagram of AAO substrate, carbon nitride nanotubes and molecular structure of the carbon nitride nanotubes.

Graphitic carbon nitride (g-CN) was chosen as it is formed from tri-*s*-triazine moieties interconnected via tertiary amines in a well-defined way without doping or modification, composed of only the two earth-abundant elements carbon and nitrogen. Moreover, it meets our requirements to fabricate negatively charged carbon nitride nanotubes and a fully condensed conjugated structure that stabilizes the π-electron system for a high charge mobility [25]. The g-CN nanotube membrane (CNNM) was fabricated through vapor deposition–polymerization of melamine as the starting material (Figure 1a), which is a common way to obtain g-CN [25–27]. In this work, melamine and the AAO membrane with a pore diameter of 100 nm were placed in a tube furnace with N_2_ flow (Figure 1b) [16]. The evaporation temperature was set to 573 K while the polymerization temperature was set to 773 K. Then, the evaporated precursor was deposited and polymerized in the porous AAO membrane, generating carbon nitride nanotubes. Figure 1c shows a schematic of a bare AAO membrane and the carbon nitride nanotubes formed in the AAO membrane. Similar to bulk g-CN fabricated by thermally induced polycondensation, the CNNM has a planar one-dimensional molecular structure based on NH-bridged tri-*s*-triazine units [25]. Meanwhile, the diameter of CNNMs can be well adjusted by controlling the amount of precursor (Figure S1, Supporting Information File 1). CNNMs fabricated by this approach possess a high nitrogen content with excellent chemical and thermal stability (Figures S2 and S3, Supporting Information File 1). It is also environmentally friendly, sustainable, and can be facilely synthesized on large scales with low cost [28–29].

**Figure 2:**
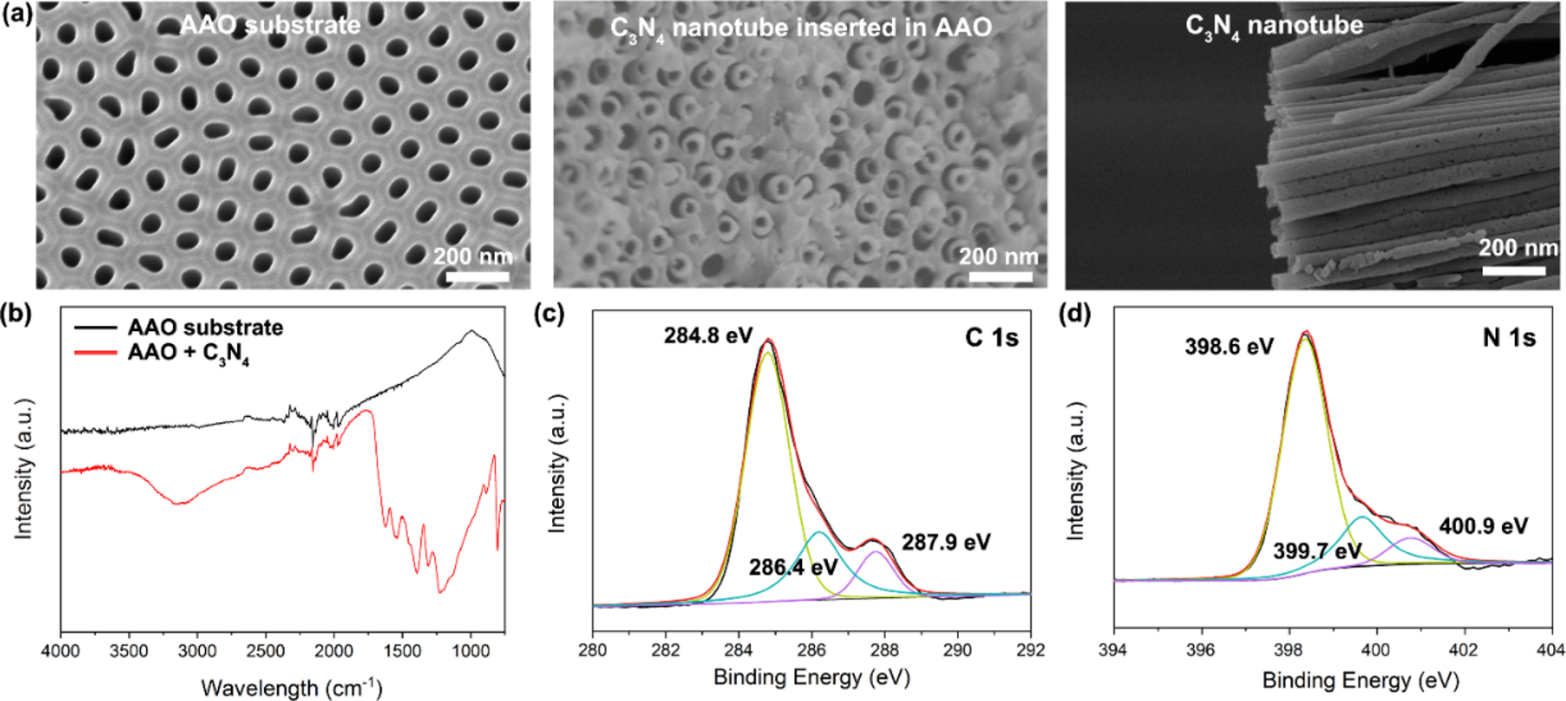
Carbon nitride nanotube properties. (a) SEM images of AAO substrate, carbon nitride nanotubes grown in the AAO substrate after polymerization, and carbon nitride nanotubes after removing the AAO substrate. (b) FTIR spectra before and after polymerization. (c) High-resolution XPS C 1s spectra of carbon nitride nanotubes, indicating two typical C 1s peaks at 284.8 and 287.9 eV. The former can be assigned to the sp^2^-hybridized carbon atoms in the N-containing aromatic ring, while the latter is typically assigned to impurity carbon, such as graphitic C or grease. (d) High-resolution XPS N 1s spectra that are deconvoluted into three peaks, 398.6 eV (C=N–C), 399.7 eV (N–C_3_), and 400.9 eV (C–NH–C and C–NH_2_).

Figure 2a shows the typical SEM image of the AAO membrane, CNNM@AAO, and the CNNM. After polymerization, there are carbon nitride nanotubes grown on the walls of the AAO nanochannels. The AAO substrate can be removed by immersion in 1 M hydrochloric acid to obtain the free-standing CNNM. The carbon nitride nanotubes have an external diameter of about 100 nm, and an inner diameter of about 60 nm. The chemical structure of carbon nitride nanotubes was further analyzed using FTIR (Figure 2b). The bare AAO substrate showed no obvious absorption peaks, while CNNM@AAO showed broad peaks between 3500 and 3000 cm^−1^, which originate from the terminal amino groups. The typical stretching modes of CN heterocycles were found at 1200 to 1600 cm^−1^, and the characteristic breathing mode of the triazine units was found at approximately 800 cm^−1^ after polymerization, thus indicating the formation of carbon nitride nanotubes [30–32].

Beyond that, high-resolution XPS spectra of C 1s and N 1s were illustrated in Figure 2c and Figure 2d, which further confirm the tri-*s*-triazine-based carbon nitride structure. The C 1s spectra can be deconvoluted into three peaks centered at 284.8, 286.4, and 287.9 eV, while N 1s spectrum can be deconvoluted into three peaks at 398.6, 399.7, and 400.9 eV. The C 1s peaks at 286.4 and 287.9 eV are associated to the major aromatic carbon species in the graphitic carbon nitride framework, representing the sp^2^-hybridized carbon atoms in the N-containing aromatic ring. The N 1s peak in 398.6 eV is from the sp^2^-hybridized nitrogen in the tri-*s*-triazine rings. The peak at 399.7 eV indicates the tertiary nitrogen N–C_3_ groups. In addition, the terminal amino groups on the surface are also confirmed by the peak at 400.9 eV. All these results are consistent with graphitic carbon nitride powder reported before [28,33] indicating the formation of carbon nitride nanotubes.

### Ion transport in carbon nitride nanotube membrane

**Figure 3:**
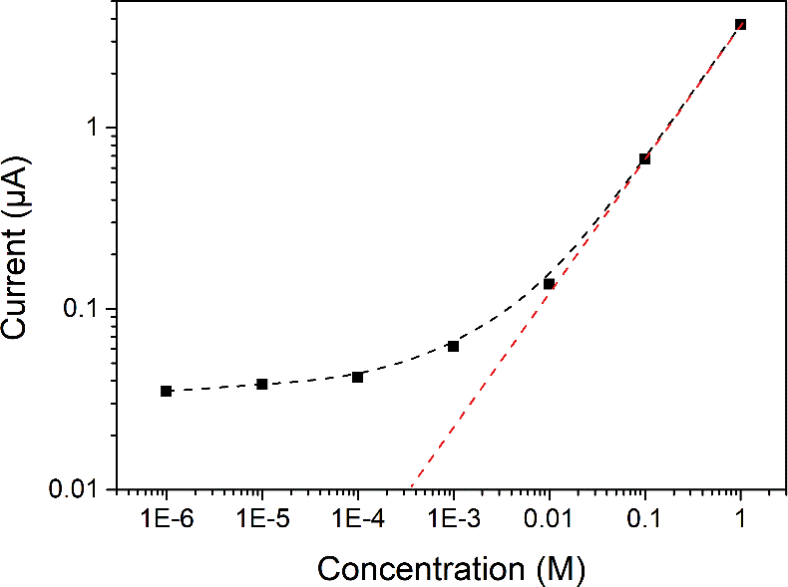
Conductance as a function of the salt concentration, indicating ion transportation controlled by surface charges in carbon nitride nanotubes (black squares). The red curve shows the data of the bulk solution (red curve).

As reported before [34–35], the graphitic carbon nitride fabricated by thermal polymerization has a negative surface charge in the initial state because of the incomplete polymerization or condensation with electron-rich –NH terminal groups. The negative surface charge is a crucial factor in ion transport. To confirm that confinement effects as well as the surface charge control the ion-transport properties [36–38], we measured the conductance of KCl electrolyte both in bulk solution and across the carbon nitride nanotubes (Figure S4, Supporting Information File 1). Figure 3 showed the conductance as a function of salt concentration (KCl) both in bulk solution and across the CNNM. The conductivity of the bulk solution is proportional to the concentration of KCl, while the ionic conductance across the carbon nitride nanotubes remarkably deviates from the bulk value of 0.1 M to 10^−6^ M with a leveling of the current at approximately 0.35 µA. This indicates that the ion transport through the CNNM is fully governed by surface charges, which provides a possibility to control the ion transport by CNNMs.

### Modification of the carbon nitride nanotube membrane

**Figure 4:**
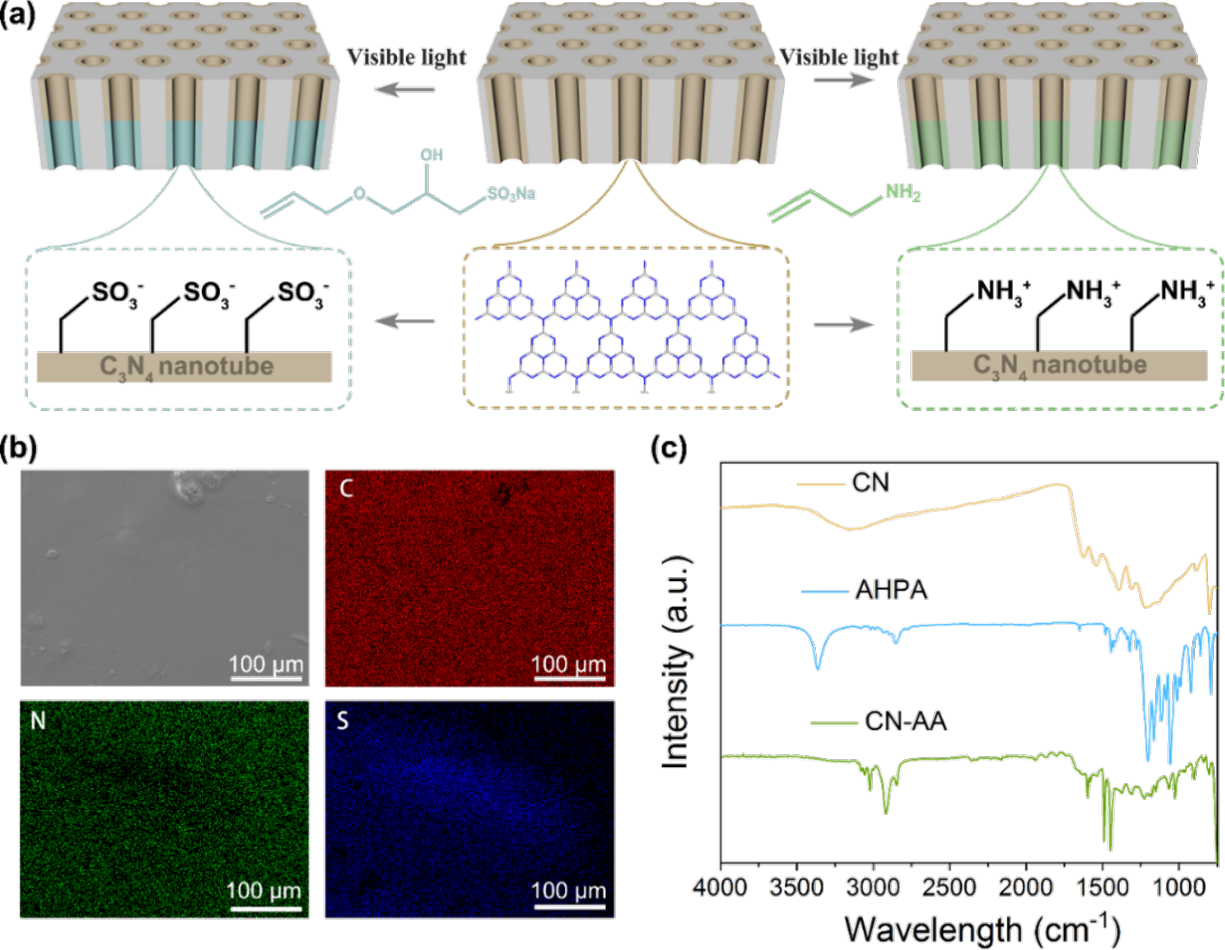
(a) Scheme of the unilateral modification process and surface properties after modification. (b) The SEM view and corresponding elemental mapping (C, N, S) of CNNM after unilateral AHPA group modification. (c) FTIR spectra before and after the unilateral modification with AHPA and AA groups.

The current–voltage (*I*–*V*) measurements of the bare carbon nitride nanotubes showed that ion transport across the CNNM is symmetric. The CNNM shows no ion rectification because of its symmetric structure and surface-charge distribution (Figure S5, Supporting Information File 1). In order to obtain rectified ion transport, we modified the carbon nitride nanotubes unilaterally with a solution of 3-allyloxy-2-hydroxy-1-propanesulfonic acid sodium salt (AHPA) and with allylamine (AA) (Figure 4a). As reported before [39–40], radicals can be created on the surface of g-CN through irradiation with visible light and different molecules can then be grafted to integrate various functionalities. The modification of CNNM with AHPA was confirmed by elemental mapping. As shown in Figure 4b, elemental mapping of the AHPA-modified membrane clearly shows the existence of sulfur atoms on the surface while unmodified side only contains carbon and nitrogen (Figure S6, Supporting Information File 1). The AHPA modification was also confirmed by FTIR spectra recorded before and after modification (Figure 4c). After modification, there is an obvious peak near 2950 cm^−1^, which corresponds to the C–H bond stretching, originating from grafted AHPA molecules. In the case of the AA-modified membrane, FTIR spectra showed similar phenomena before and after modification (Figure 4c). The obtained results showed that AHPA and AA molecules are grafted successfully onto the CNNM.

### Ion diode based on the modified carbon nitride nanotube membrane

**Figure 5:**
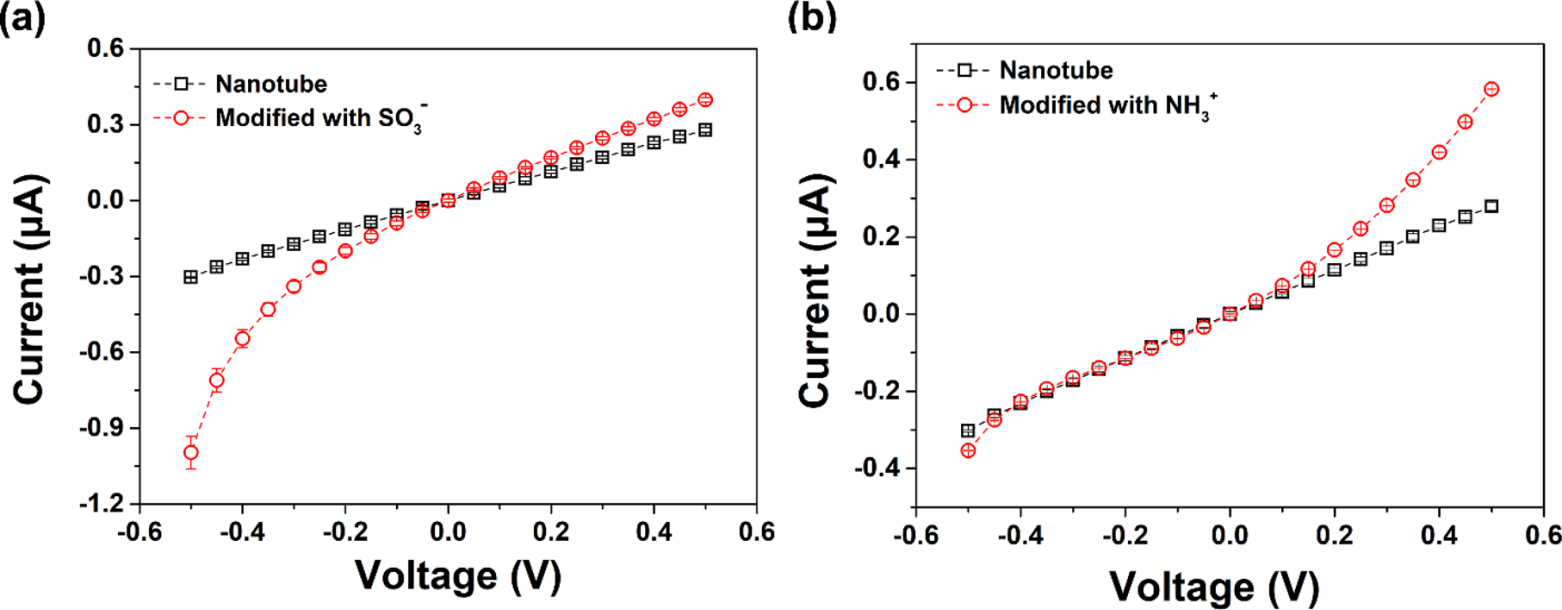
(a) Current–voltage curves before and after unilateral modification with AHPA. (b) Current–voltage curves before and after unilateral modification with AA.

Figure 5a shows the *I*–*V* curves before and after unilateral functionalization with AHPA. Before modification, the *I*–*V* curve is symmetric; while after AHPA modification, the ionic current at −0.5 V increased from −0.3 to −1.0 μA and the ionic current at +0.5 V only increased from 0.3 to 0.42 μA. This means the AHPA-modified CNNM shows an obvious ion rectification due to the unilateral introduction of negatively charged groups. Analogously, the AA-modified CNNM also showed asymmetric ion transport. The ionic current at +0.5 V increased from 0.3 μA to 0.6 μA while ionic current at −0.5 V did not change after modification (Figure 5b). The asymmetric ion transport can be ascribed to the asymmetric surface distribution of negatively charged amino groups. Overall, the deviation from the reference material is shifted towards negative values in the case of AHPA modification and towards positive values in the case of AA modification. This can be attributed to the incorporation of oppositely charged functional groups. Thus, it is possible to tailor the direction of ion rectification via the photofunctionalization process and the choice of grafted molecules.

## Conclusion

We fabricated a carbon nitride nanotube membrane by simple vapor deposition–polymerization and modified it via a photo-induced functionalization process to alter the ion transport properties. The carbon nitride nanotube membrane showed ion transport that was governed by surface charges of the electron-rich –NH terminal groups. After unilateral modification with AHPA and AA molecules, the symmetric surface charge distribution was altered, leading to ion rectification in the CNNM. With the advantages of low-cost, thermal and mechanical robustness, and simple fabrication process, the CNNM with ion rectification has the potential to be used in the energy generation through salinity gradients and ion sensor system.

## Experimental

**Materials:** Unless otherwise noted, all of the commercial reagents were used as received. Allylamine (98%, Sigma-Aldrich), 3-allyloxy-2-hydroxy-1-propanesulfonic acid sodium salt solution (40 wt %, AHPA, Sigma-Aldrich) and melamine (purity >98.0%, Sigma-Aldrich). 60 μm thick AAO membranes with a pore width of 84 ± 16 nm were purchased from Heifei Puyuan Nano, China. Glass test tubes for vapor deposition–polymerization (VDP) were purchased from Merck Millipore.

**Characterizations:** The released CNNM was transferred to a quartz glass substrate and analyzed. X-ray photoelectron spectroscopy (XPS) was performed by an ESCALab220i-XL electron spectrometer from VG Scientific using 300W Al Kα radiation, while the base pressure was about 3 × 10^−9^ mbar. The binding energies were referenced to the C 1s line at 284.8 eV from adventitious carbon. A scanning electron microscope (SEM) JSM-7500F (JEOL) at an accelerating voltage of 3 kV was used to get the top view of the CNNs. X-ray diffraction (XRD) patterns were recorded with a Bruker D8 Advance instrument with Cu Kα radiation. Fourier transform infrared (FTIR) spectra were recorded with a Thermo Scientific Nicolet iS5 FTIR spectrometer.

**Fabrication of CNNM:** The carbon nitride nanotube membrane was fabricated by a VDP method described before [16]. Firstly, the commercial AAO membrane (diameter: 5 mm) was cleaned with ethanol and deionized water, then dried with nitrogen. Subsequently, the cleaned AAO and the precursor melamine were put on the bottom of the glass test tube. The samples were placed in the oven to heat to 773 K with a heating rate of 10 K/min, and then kept for 4 h to ensure polymerization. After the temperature naturally cooled down to ambient temperature, the AAO membrane turned from transparent white to brown, and yellowish carbon nitride power at the bottom of the test tube was obtained. To get a pure carbon nitride nanotube for TEM or SEM, the carbon nitride nanotube membrane was immersed in 1 M acid for chemical etching (72 h), then cleaned by deionized water and dried in an oven at 60 °C.

**Modification:** In a similar manner as described in our previous paper [39], 1 g of AHPA solution (40 wt % in water) and 1 g of deionized water were mixed (or 1 g of allylamine and 1 g of ethanol). The mixture was sonicated for 10 min, and nitrogen was flushed through the mixture for 3 min for the removal of dissolved oxygen. The CNNM was placed in a glass dish, then AHPA or AA solution was dropped onto the surface of the CNNM. The mixture was irradiated by 50 W LED daylight sources for the desired reaction time. Afterward, the mixture was vacuum-filtered, washed three times with water (3 × 50 mL), and washed once with acetone (20 mL).

**Ion diode measurements:** The setup for the measurement of the ion diode properties is shown in Figure S3 (Supporting Information File 1). The membrane was caught in a H-cell with electrolyte. A Ag/AgCl electrode was used to collect the ionic current. The *I*–*V* curves were adjusted to zero current at zero voltage to remove small offsets experienced between runs. All measurements were carried out at ambient temperature. The main transmembrane potential used in this work was stepped from −0.5 to +0.5 V at 0.05 V/step with 1 s/step (0.05 V/s). CNNMs before and after modification were mounted between two chambers of a custom-made H cell, which was filled with electrolyte. Ag/AgCl electrodes were used to collect the current and voltage signals. The ionic current was measured with a Keithley 6430 picoamperemeter (Keithley Instruments, Cleveland, OH).

## Supporting Information

File 1 Additional experimental data.
