# Supplementary material for: A biomimetic nanofluidic diode based on surface-modified polymeric carbon nitride nanotubes
Source: Beilstein J Nanotechnol. 2019 Jun 27;10:1316–23. doi: 10.3762/bjnano.10.130 (PMC6604718; doi:10.3762/bjnano.10.130)
Supplement: File 1 — Additional experimental data. [file Beilstein_J_Nanotechnol-10-1316-s001.pdf]

BEILSTEIN JOURNAL OF NANOTECHNOLOGY

Supporting Information

for

A biomimetic nanofluidic diode based on surface-modified polymeric carbon nitride nanotubes

Kai Xiao, Baris Kumru, Lu Chen, Lei Jiang, Bernhard V. K. J. Schmidt and Markus Antonietti

Beilstein J. Nanotechnol. **2019,** *10,* 1316–1323. doi:10.3762/bjnano.10.130

Additional experimental data

License and Terms: This is a supporting information file under the terms of the Creative Commons Attribution License (http://creativecommons.org/licenses/by/4.0). Please note that the reuse, redistribution and reproduction in particular requires that the authors and source are credited. The license is subject to the *Beilstein Journal of Nanotechnology* terms and conditions: (https://www.beilstein-journals.org/bjnano)

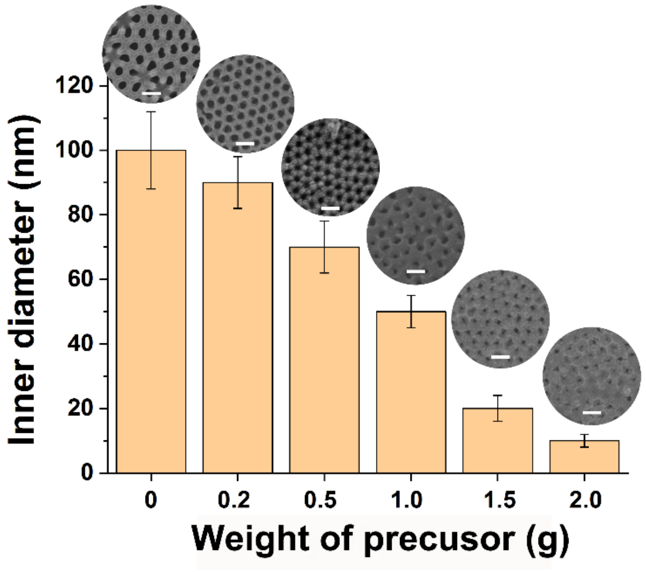

Figure S1: The SEM images of the carbon nitride nanotube membrane with diameters of 100 nm (bare AAO), 90 nm, 70 nm, 50 nm, 20 nm, and 10 nm. Scale bar 200 nm.

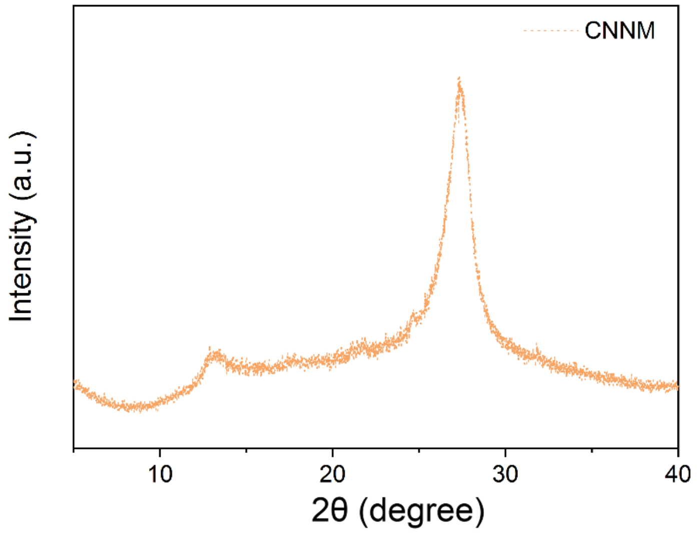

Figure S2: XRD of CNNM.

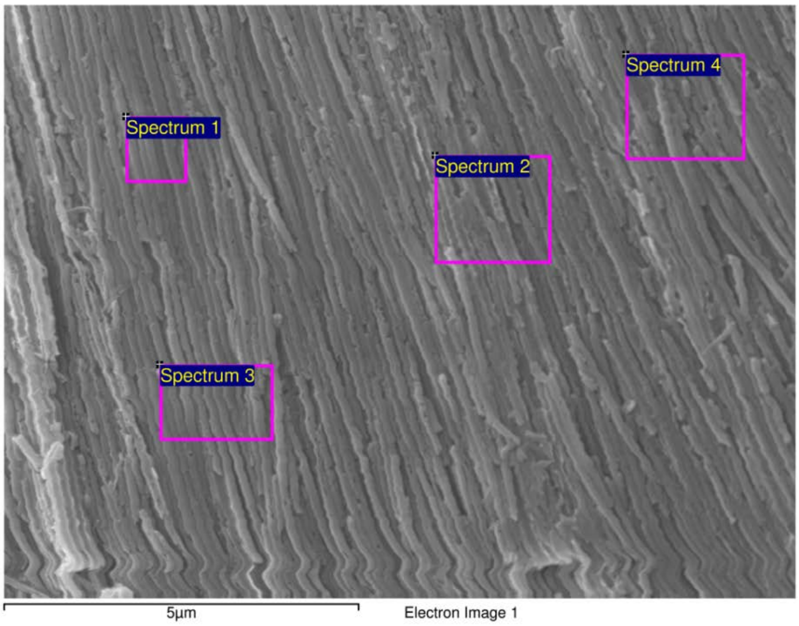

Processing option : All elements analysed (Normalised)

Spectrum	In stats.	C	N	Total
Spectrum 1	Yes	43.64	56.36	100.00
Spectrum 2	Yes	37.63	62.37	100.00
Spectrum 3	Yes	43.29	56.71	100.00
Spectrum 4	Yes	40.44	59.56	100.00
Mean		41.25	58.75	100.00
Std. deviation		2.81	2.81	
Max.		43.64	62.37	
Min.		37.63	56.36	

All results in weight%

Figure S3: SEM image and corresponding elemental content of CNNM.

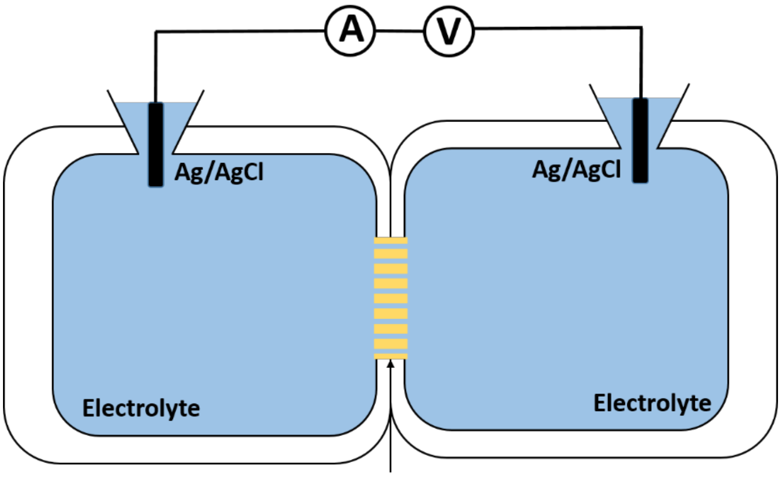

Figure S4: Schematic test setup.

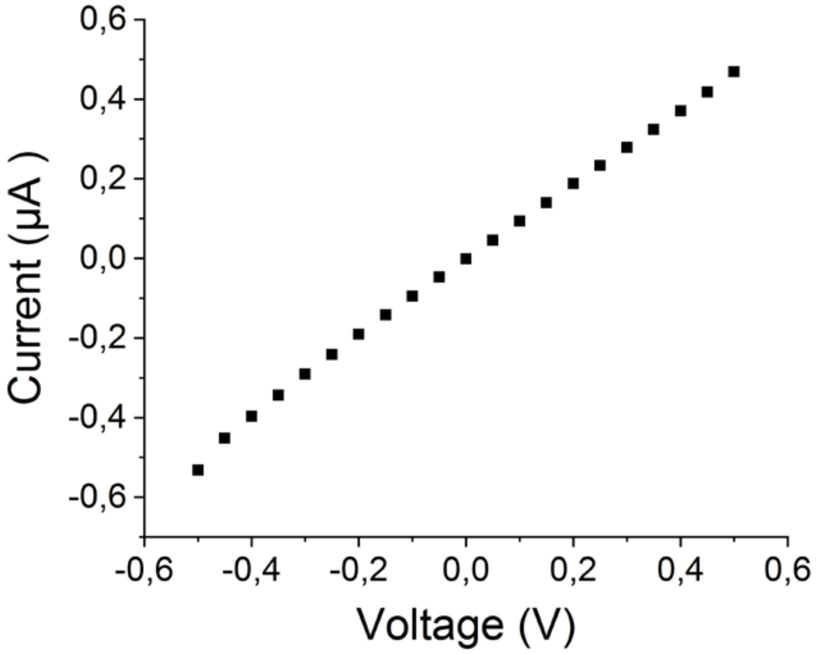

Figure S5: Symmetric current–voltage curve of bare carbon-nitride nanotube membrane.

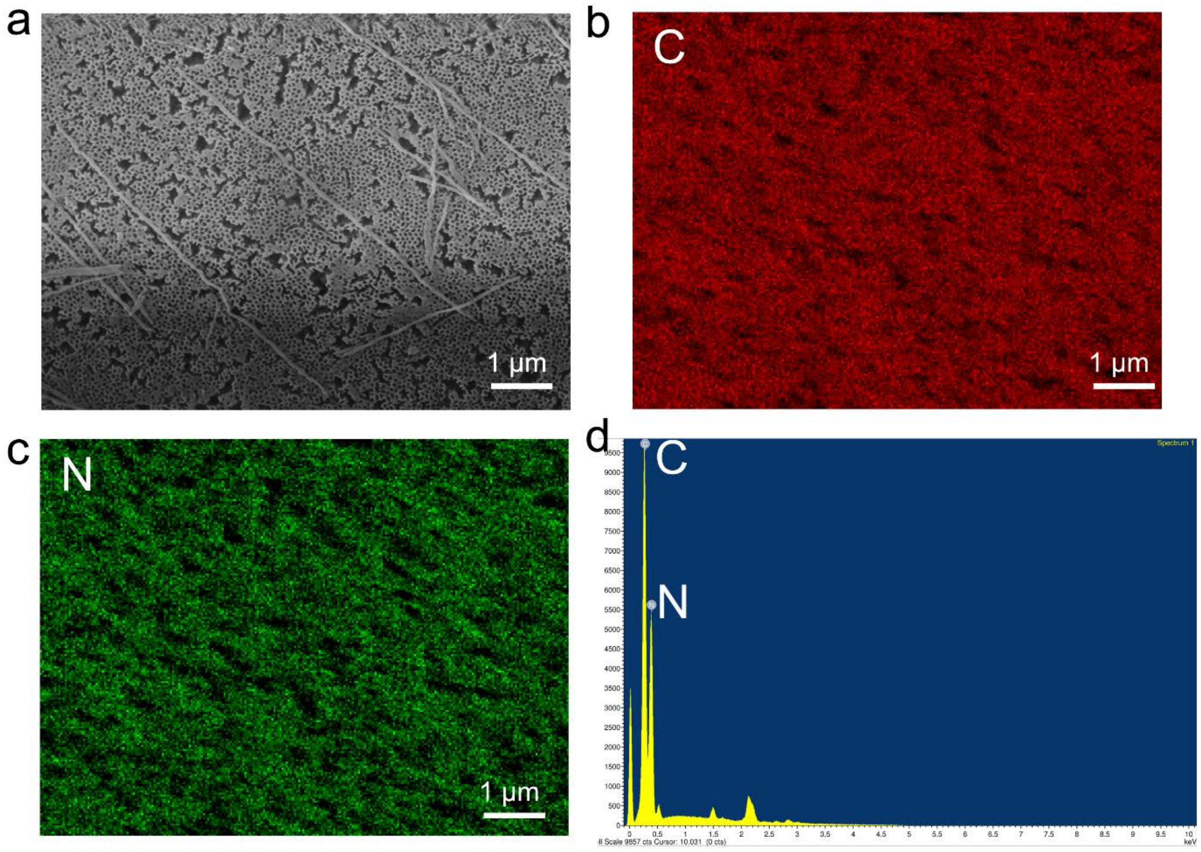

Figure S6: SEM images and corresponding elemental content of CNNM.
